# The complete mitochondrial genome of *Ctenochaetus flavicauda* (Acanthuriformes, Acanthuridae)

**DOI:** 10.1080/23802359.2020.1719923

**Published:** 2020-02-02

**Authors:** Liguo Yang, Yue Wang, Na Ying, Xuefeng Song, Yanqing Wu, Bo Qin

**Affiliations:** a Key Laboratory of East China Sea Fishery Resources Exploitation, Ministry of Agriculture, East China Sea Fisheries Research Institute, Chinese Academy of Fishery Sciences, Shanghai, China;; b Guangdong Provincial Key Laboratory of Marine Biotechnology, Institute of Marine Sciences, Shantou University, Shantou, Guangdong, China

**Keywords:** *Ctenochaetus flavicauda*, Acanthuridae, mitochondrial genome

## Abstract

*Ctenochaetus flavicauda* is one of most important genera of Acanthuridae. However, the systemically classification and taxonomic studies have so far been limited. In this study, we report the complete mitochondrial genome sequence of *C*. *flavicauda*. The mitogenome has 15,772 base pairs (55.5% A + T content) and made up of total of 37 genes (13 protein-coding, 22 transfer RNAs and 2 ribosomal RNAs), and a putative control region. This study will provide useful genetic information for future phylogenetic and taxonomic classification of Acanthuridae.

*Ctenochaetus flavicauda* belongs to the Family Acanthuridae and the Order Acanthuriformes, *C. flavicauda* is the smallest of the Ctenochaetus. They grow to a maximum length of around 16 centimeters. This species has a juvenile dress. The young animals have a bright yellow color and have blue eyes. As soon as they grow up they get a red/brown color with orange dots on the head that change into horizontal stripes on the flank (Randall et al., 2001).

There is no report of the complete genome of this species *C. flavicauda*, which was developed in Shenzhen, Guangdong Province, Republic of China (N22°37′34″, E114°41′06″) in October 2019. Therefore, it is very important to characterize the complete mitogenome of this species, which can be utilized in research on taxonomic resolution, population genetic structure and phylogeography, and phylogenetic relationship. Total DNA was extracted from muscle following TIANamp Marine Animals DNA Kit (Tiangen, China), and NOVOPlasty software was used to assemble the mitogenomes, the mistake parameter was set by default (Dierckxsens et al., 2017). The samples were stored in −80 °C in Key Lab of South China Sea Fishery Resources Exploitation & Utilization, Ministry of Agriculture and Rural Affairs, South China Sea Fisheries Research Institute, Chinese Academy of Fishery Sciences, Guangzhou, China. Number is CF-1.

In this study, we obtained the complete mitochondrial genome of the *C. flavicauda*. Its mitochondrial genome has been deposited in the GenBank under accession number MN872234. For a better understanding of genetic status and the evolutionary study, we focused on the genetic information contained in the complete mitochondrial genomes of the fish.

The complete mitogenome of the *C. flavicauda* was 15,772 bp in length. The genomic organization was identical to those of typical vertebrate mitochondrial genomes, including two rRNA genes, 13 protein-coding genes, 22 tRNA genes, a light-strand replication origin (OL). The overall base composition was 29% of A, 26.5% of T, 28.7% of C, and 15.8% of G with a slight A + T bias (55.5%) like other vertebrate mitochondrial genomes. The features mentioned above were accordant with typical Acanthuridae fish mitogenome.

For the 13 protein-coding genes, all genes started with ATG. This feature was common among vertebrate mitochondrial protein-coding genes. *C. flavicauda* had one non-coding regions, the L-strand replication origin region (36 bp) locating between tRNA-Asn and tRNA-Cys. Except for eight tRNA (tRNA-Ser, tRNA-Pro, tRNA-Glu, tRNA-Tyr, tRNA-Cys, tRNA-Asn, tRNA-Ala, and tRNA-Gln) and the *ND6* gene were encoded on the L-strand, the others were encoded on the H-strand. This feature is similar to other fish mitochondrial genes. The complete mitogenome sequence had 16s RNA (1,691 bp) and 12s RNA (951 bp), which were located between tRNA-Phe and tRNA-Leu and separated by tRNA-Val gene. The location is same with most vertebrates that have high conservation.

To determine taxonomic status of *C. flavicauda*, we reconstructed the phylogeny of this species with other natural populations based on the *COI* gene. The phylogenetic tree showed that the *C. flavicauda* has the closer relationship with *Ctenochaetus striatus* (Figure 1). The phylogeny was reconstructed based on the General Time Reversible + Invariant + gamma sites (GTR + I+ G) model of nucleotide substitution using Mega7 (Kumar et al., 2016). The complete mitochondrial genome sequence of the *C. flavicauda* provided an important dataset for a better understanding of the mitogenomic diversities and evolution in fish as well as novel genetic markers for studying population genetics and species identification.

**Figure 1.**
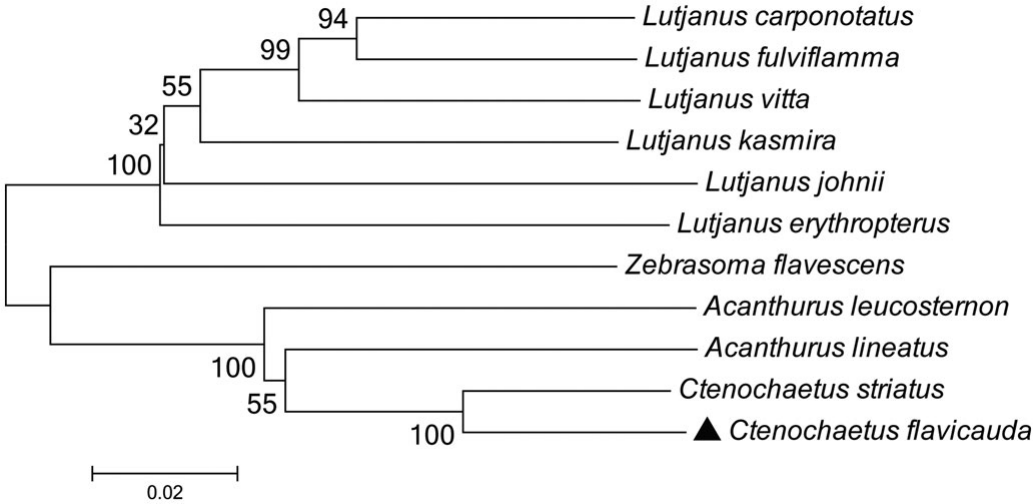
The phylogenetic relationship was estimated using the maximum-likelihood method for the *COI* genes. Genbank accession Numbers: *Ctenochaetus striatus* (KU244260), *Acanthurus lineatus* (EU273284), *Acanthurus leucosternon* (EU136032), *Lutjanus carponotatus* (NC_044104), *Zebrasoma flavescens* (AP006032), *Lutjanus kasmira* (FJ416614), *Lutjanus fulviflamma* (NC_043916), *Lutjanus vitta* (NC_042930), *Lutjanus erythropterus* (KP939271), *Lutjanus johnii* (KJ643926), and *Ctenochaetus flavicauda* (MN872234). The numbers at the nodes are bootstrap percent probability values based on 1000 replications.
